# An efficient double-fluorescence approach for generating *fiber-2*-edited recombinant serotype 4 fowl adenovirus expressing foreign gene

**DOI:** 10.3389/fmicb.2023.1160031

**Published:** 2023-03-31

**Authors:** Yiwen Guo, Zhenqi Xu, Yifei Chao, Xudong Cao, Huiru Jiang, Han Li, Tuofan Li, Zhimin Wan, Hongxia Shao, Aijian Qin, Quan Xie, Jianqiang Ye

**Affiliations:** ^1^Key Laboratory of Jiangsu Preventive Veterinary Medicine, Key Laboratory for Avian Preventive Medicine, Ministry of Education, College of Veterinary Medicine, Yangzhou University, Yangzhou, Jiangsu, China; ^2^Jiangsu Co-Innovation Center for Prevention and Control of Important Animal Infectious Diseases and Zoonoses, Yangzhou, Jiangsu, China; ^3^Joint International Research Laboratory of Agriculture and Agri-Product Safety, The Ministry of Education of China, Yangzhou University, Yangzhou, Jiangsu, China; ^4^Institutes of Agricultural Science and Technology Development, Yangzhou University, Yangzhou, Jiangsu, China

**Keywords:** recombinant FAdV-4, *fiber-2*, CRISPR/Cas9, Cre-LoxP, foreign gene, vaccine

## Abstract

Recently, the infection of serotype 4 fowl adenovirus (FAdV-4) in chicken flocks has become endemic in China, which greatly threatens the sustainable development of poultry industry. The development of recombinant FAdV-4 expressing foreign genes is an efficient strategy for controlling both FAdV-4 and other important poultry pathogens. Previous reverse genetic technique for generating the recombinant fowl adenovirus is generally inefficient. In this study, a recombinant FAdV-4 expressing enhanced green fluorescence protein (EGFP), FA4-EGFP, was used as a template virus and directly edited *fiber-2* gene to develop an efficient double-fluorescence approach to generate recombinant FAdV-4 through CRISPR/Cas9 and Cre-Loxp system. Moreover, using this strategy, a recombinant virus FAdV4-HA(H9) stably expressing the HA gene of H9N2 influenza virus was generated. Chicken infection study revealed that the recombinant virus FAdV4-HA(H9) was attenuated, and could induce haemagglutination inhibition (HI) titer against H9N2 influenza virus at early time points and inhibit the viral replication in oropharynx. All these demonstrate that the novel strategy for constructing recombinant FAdV-4 expressing foreign genes developed here paves the way for rapidly developing attenuated FAdV-4-based recombinant vaccines for fighting the diseases caused by both FAdV-4 and other pathogens.

## Introduction

Adenoviruses are non-enveloped, double-stranded DNA viruses, and are further divided into six genera: *Atadenovirus, Aviadenovirus, Ichtadenovirus, Mastadenovirus, Siadenovirus*, and *Testadenovirus* (Benko et al., 2022). Fowl adenoviruses (FAdVs) belong to the family of Adenoviridae and the genus of *Aviadenovirus*, and are defined as 5 species (A-E) with 12 serotypes (1–7, 8a, 8b, and 9–11) (Steer et al., 2009; Marek et al., 2010). Epidemiological data revealed that hepatitis-hydropericardium syndrome (HHS) caused by serotype 4 fowl adenovirus (FAdV-4) has been endemic in China since 2015, and resulted in huge economic loss to poultry industry. Multiple-vaccine against FAdV-4 and other important poultry pathogens are urgently needed. Development of recombinant FAdV-4 expressing foreign genes is an ideal approach for efficiently controlling both FAdV-4 and other poultry viruses.

Human adenovirus type 5 (HAdV-5) has been extensively studied as a vector for gene delivery (Sharma et al., 2009), while FAdV is relatively less studied. Recent studies showed that FAdV has also been used as a vector to express exogenous genes, including VP2 of IBDV (Infectious Bursal Disease Virus), S1 of IBV (Infectious Bronchitis Virus) and Fiber of FAdV-8b (Sheppard et al., 1998; Johnson et al., 2003; Francois et al., 2004; Pan et al., 2021; Lu et al., 2022; Wang et al., 2022). However, the approaches used to generate recombinant FAdV in these studies mainly depend on the use of infectious clones or traditional homologous recombination, and was time-consuming and inefficient, particularly for the cloning of the large genome of FAdV and for the fussy procedure of the screen and purification of the recombinant viruses. In our previous studies, we used CRISPR/Cas9 technique and enhanced green fluorescence protein (EGFP) reporter to efficiently generate two *fiber-2*-edited attenuated recombinant FAdV-4 viruses, FA4-EGFP and FAdV4-EGFP-rF2 (Xie et al., 2021, 2022). To develop a highly efficient approach to construct attenuated recombinant FAdV-4 expressing foreign gene without reporter, in this study, we used FA4-EGFP as a template virus to replace wild type FAdV-4, and combined CRISPR/Cas9 and Cre-Loxp system to directly edit *fiber-2* gene to generate recombinant FAdV-4.

Although the inactivated vaccine against H9N2 avian influenza virus (AIV) has been widely implemented in China, H9N2 viruses have been frequently isolated from the vaccinated chicken flocks (Gu et al., 2017; Liu Y. et al., 2020), highlighting the shortcomings of the current vaccines against H9N2. To prove the strategy for generating recombinant FAdV-4 expressing foreign gene, and generate a live vaccine candidate for better protection against H9N2, we developed a novel attenuated recombinant virus FAdV4-HA(H9) expressing the hemagglutinin (HA) gene of H9N2 for vaccine candidate against both FAdV-4 and H9N2.

## Materials and methods

### Cells, viruses, and antibodies

The highly pathogenic FAdV-4 strain SD2015 was isolated from the liver of an infected chicken during hepatitis-hydropericardium syndrome outbreak in Shandong province (Ye et al., 2016). The recombinant virus FA4-EGFP with EGFP fused with Fiber-2 was constructed in our laboratory (Xie et al., 2021). Fowl adenoviruses were cultured in LMH cells from ATCC and maintained in DMEM/F12 (Gibco, NY, USA) containing 10% fetal bovine serum (Lonsera, Shanghai, China). A/chicken/Xuzhou/491/2019(H9N2) (XZ491) virus was kept in our laboratory (Wan et al., 2023). Monoclonal antibodies (mAb) 2G10 against HA of H9N2, mAb 3B5 against Fiber-1 of FAdV-4 and positive chicken serum against FAdV-4 were generated and stored in our laboratory (Shao et al., 2019). PE AffiniPure F(ab’)2 Fragment goat anti-mouse IgG (H + L) was purchased from Yeason (Shanghai, China). The AceQ qPCR SYBR Green Master Mix was purchased from Vazyme (Nanjing, China).

### Design of sgRNAs and donor plasmids

The sgRNAs targeting Fiber-2 of FAdV-4 were designed using the guide RNA designing website,^1^ introduced with *Bsm*BI restriction sites, and cloned into the sgRNA expression vector lentiCRISPR v2. To construct the donor plasmid, the two consecutive Loxp sequences were firstly synthesized and cloned into the pUC57 vector. The RFP gene with poly(A) signal under the promoter of EF-1α was then amplified, and cloned into the vector between the two loxp sequence. Then, the Fiber-2 of FAdV-4, flanked with 1 kb left homologous arm and 1 kb right homologous arm at both sides, after introduced with sgRNA recognition sequence at both ends, was amplified and cloned into pMD19 vector (Takara, Dalian, China). Subsequently, the RFP expression cassette flanked with loxp sequence was amplified, and cloned into the site between the *fiber-2* and right homologous arm through one-step cloning. The primers used for guide RNA cloning and constructing donor plasmid are listed in Tables 1, 2, respectively.

**TABLE 1:** List of primers for sgRNA cloning.

Sequence of primers (5′–3′)	
sgRNA	F: CACCGAAGGGTGTATCGCTCTCCGG
	R: AAACCCGGAGAGCGATACACCCTTC

**TABLE 2:** List of primers for constructing donor plasmid.

PCR products	Sequence of primers (5′–3′)
RFP expression cassette	F: GAATTGGCTCCGGTGCCCGTCAGT
	R: CATGGTGGCAGCGCTCTAGAACCGGTCCTGTGTTC
Fiber-2 + left and right homologous arm	F: GTGTATCGCTCTCCGGAGGGGTGACCTACTGACCCTC
	R: GGTGTATCGCTCTCCGGAGGTTCGTGGAACGCTCCGTCAG
RFP expression cassette flanked with loxp	F: TAACTCGAGTCTAGAAAGCTTGGATC
	R: GGGAGGGCGCGCATAACTTCGTATAATGTATGCTATACGAAGTTATGGTACCGATATC
HA of H9N2	F: CCCATCATCATCAAGAACGAGACAGTATCACTAATAAC
	R: CTTTCTAGACTCGAGTTATATACAAATGTTGCATC

### Generation of the recombinant FAdV4-HA(H9)

LMH seeded in 6-well plate at a confluency of 80% were co-transfected with 2 μg of sgRNA and donor plasmid using TransIT-LT1 (MIRUS, WI, USA) according to the manufacturer’s instructions. At 10 hours (h) post transfection, the LMH cells were infected with 0.1 multiplicity of infection (MOI) of FA4-EGFP. At 48-72 h post infection (hpi), the supernatants of infected LMH cells were harvested and centrifuged at 12,000 rpm, and subsequently inoculated into the fresh LMH cells prepared the day before harvest. Fluorescence was then observed daily, and RFP was used as an indicator of recombinant virus to differ from the parental virus which expressing EGFP. The recombinant virus, expressing the HA of H9N2 AIV and red fluorescence, was selected and purified through limiting dilution and virus plaque pickup. To remove the RFP expression cassette, the LMH cells in 6-well plate was transfected with 4 μg of pcDNA3.1-Cre, and subsequently infected with 100 TCID_50_ of recombinant virus. At 48–72 hpi, the RFP-negative plaques were selected and serially diluted, and subsequently inoculated into fresh LMH cells for further purification. The excision of RFP expression cassette from the recombinant virus was confirmed by PCR and sequencing. The primers used for identification of recombinant virus are listed in Table 3.

**TABLE 3:** List of primers for detecting recombinant virus.

PCR products	Sequence of primers (5′–3′)
31636-33285	F: CTCCAACTGGTTTGACCAGAACG
	R: GTTGTATGATTGGACGCGGGAAC

### Western blot analysis

The LMH cells were seeded in 12-well plate the day before infection. The recombinant virus with 0.1 MOI was then inoculated into the LMH cells. At 48 hpi, the LMH cells and supernatants were harvested, the cells were then washed once with PBS buffer, and subsequently lysed in 100 μl lysis buffer with proteolytic protease and phosphatase inhibitor cocktail (NCM, Soochow, China). The lysates were then centrifuged at 12,000 rpm for 10 min to remove debris, the supernatants of lysates or supernatants collected from the 12-well plate were then boiled with protein loading buffer (Solarbio, Beijing, China). The samples were separated on a 10% SDS-PAGE gel and transferred onto nitrocellulose membranes (Cytiva, MA, USA). The membranes were then blocked with NCMblot blocking buffer (NCM, Soochow, China) for 30 min at room temperature (RT), and incubated with corresponding antibodies. After being washed with PBST for three times, the membranes were then incubated with HRP-labeled secondary antibodies for 1 h at RT. After another three washes, the membranes were developed with chemiluminescent regents and imaged with an automatic imaging system (Tanon 5200).

### Indirect fluorescence analysis (IFA)

LMH cells seeded in 96-well plate were infected with 0.01 MOI of recombinant virus. At 48 hpi, the LMH cells were fixed with a pre-chilled acetone: ethanol (3:2 v/v) mixture for 5 min at RT. After the mixture was removed and evaporated, the cells were incubated with diluted mAb 2G10 against HA of H9N2 and chicken serum against FAdV-4 for 45 min at 37^°^C. After three washes with PBS, the cells were incubated with the diluted secondary antibodies for another 45 min at 37^°^C. After another three washes with PBS, the cells were observed by fluorescence microscopy.

### Stability of the recombinant virus

The recombinant virus was serially passaged in LMH cells for 15 passages. Expression of the HA was examined by IFA, the viral genome was extracted for PCR and sequenced every 5 passages. The primers used for PCR identification are listed in Table 3.

### *In vitro* growth curve

The LMH cells were seeded in 6-well plates the day before infection, 0.1 MOI of recombinant virus and wild-type FAdV-4 were then inoculated into the LMH cells, respectively. The supernatants were harvested at 24, 48, and 72 hpi, and stored at −80^°^C until used. The 50% tissue culture infectious doses (TCID_50_) of the harvested viruses were determined in 96-well plates by serial dilution from 10^–1^ to 10^–8^ in triplicate, and the diluted viruses were subsequently inoculated into the LMH cells. At 96 hpi, the LMH cells were fixed and examined by IFA using mAb 3B5 against Fiber-1 of FAdV-4. The fluorescence plaques after staining were analyzed and counted through fluorescence microscopy. Viral titers were then calculated by TCID_50_/ml through the Reed-Muench method (Reed and Muench, 1938).

### Animal experiment

A total of 36 specific-pathogen-free (SPF) chickens aged 2 weeks were randomly divided into three groups (*n* = 12). Chickens in group I were inoculated with the recombinant virus FAdV4-HA(H9) (Passage 10), while that in group II were inoculated with wild-type FAdV-4 (Passage 4). The chickens in group I and II were inoculated with 1 × 10^5^ TCID_50_ of indicated virus in 200 μl of culture medium intramuscularly, whereas the chickens in group III inoculated with 200 μl of culture medium intramuscularly was set as negative control. After infection, the clinical symptoms and mortality of the infected chickens were monitored daily, and blood was collected at 7, 14, and 21 day post-infection (dpi) for further serological analysis. At 21 dpi, chickens survived in group I and group III were challenged with 10^6^ TCID_50_ of XZ491 by intranasal inoculation. At 5 day post-challenge (dpc), three infected chickens per group were euthanized and oropharynx were collected to determine viral titers by SYBR Green I qRT-PCR. The animal experiments were performed in accordance with the “Guidelines for Experimental Animals”, which was approved by the Animal Care and Use Committee of Yangzhou University (Yangzhou, China). At the end of the experiment, all the chickens were euthanized by CO_2_.

### HI assay

Sera collected from the chickens were tested by HI according standard procedures. Briefly, the HA titer of the H9N2 AIV strain XZ491 was firstly titrated, and eight HA units of the XZ491 were incubated with 2-fold serially diluted (start dilution 1:5) sera at RT for 30 min, then 0.5% chicken red blood cells were added and incubated for another 30 min at RT. The HI titer was defined as the maximum dilution of serum that completely inhibited eight HA units of XZ491.

### qRT-PCR assay for viral titer in oropharynx

To determine the viral loads in the oropharynx, 200 mg of the tissue collected from the necropsied chickens were homogenized in 400 μl of PBS, then the RNA was extracted and reverse-transcribed into cDNA. The M gene of H9N2 was cloned into pMD19 vector to generate a standard plasmid and used as an indicator for the presence of the viruses. The forward and reverse primers used were 5’-GCGCAGAGACTTGAGGATGT-3’ and 5’-TGGACAAACCGTCTACGCTG-3’, respectively. The real-time qPCR experiments were performed on a Bio-Rad CFX96 qPCR system according to the manufacturer’s instructions (Vazyme, Nanjing, China). The final concentrations of viral genomes were expressed as log_10_ copy numbers per microliter of cDNA.

### Statistical analysis

All the results are presented as means ± standard deviation. The statistical analysis in this study was performed with a Student’s *t*-test using GraphPad Prism 8 software. *P* value less than 0.05 was considered significant. *, ^**^, and ^***^ indicate *P* value less than 0.05, 0.01, and 0.001, respectively.

## Results

### Novel strategy for efficient generation of the recombinant FAdV-4

**FIGURE 1:**
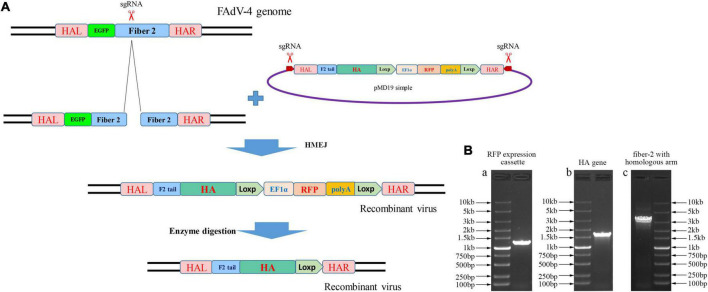
Novel strategy for generating recombinant FAdV-4. **(A)** Schematic presentation for the construction of the recombinant FAdV-4. **(B)** Construction of the donor plasmid. PCR amplification of RFP expression cassette (a), HA gene of H9N2 (b), *fiber-2* with left and right homologous arm (c).

To develop an efficient double-fluorescence approach to generate attenuated recombinant FAdV-4, CRISPR/Cas9, and Cre-Loxp system was applied by targeting the *fiber-2* gene of FAdV-4. As described in Figure 1A, sgRNA targeting the *fiber-2* of FAdV-4 and donor plasmids were co-transfected into LMH cells, followed with infection of FA4-EGFP. The recombinant virus was then rescued through homology-mediated end joining (MHEJ), and the RFP was used as an indicator of recombinant virus. After the recombinant virus was purified, the RFP expression cassette in the recombinant virus was then removed through the Cre recombinase. To generate a recombinant FAdV-4 expressing HA of H9N2, HA gene of H9N2 strain A/chicken/Xuzhou/491/2019(H9N2) (XZ491) was amplified and cloned into the site between the tail of *fiber-2* and RFP expression cassette, replacing of the shaft and knob region of *fiber-2*. As described in Figure 1B, the RFP expression cassette, HA gene of H9N2 AIV and *fiber-2* with left and right homologous arm were all efficiently amplified. The sequence of the constructed donor plasmid was confirmed by sequencing.

### The generation and purification of the recombinant virus FAdV4-HA(H9)

**FIGURE 2:**
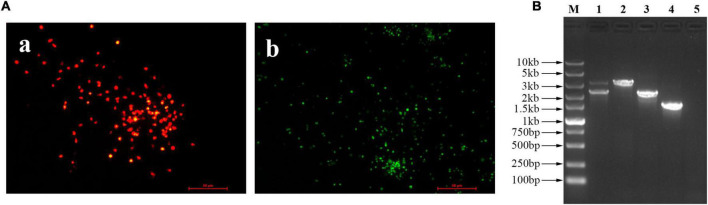
Generation and purification of the recombinant virus FAdV4-HA(H9). **(A)** The fluorescent plaques of the rescued recombinant virus FAdV4-HA(H9). At 72 hpi, the viral supernatant was blind passaged into the LMH cells, and the fluorescent plaques were observed with the fluorescent microscopy. **(a)** Red indicated the recombinant virus FAdV4-HA(H9), **(b)** green indicated the parental virus FA4-EGFP. **(B)** PCR analysis of the recombinant virus FAdV4-HA(H9). Lanes 1–5 represent unpurified recombinant virus, purified recombinant virus, parental virus FA4-EGFP, wild-type FAdV-4, and negative control, respectively. These experiments were performed twice with comparable results.

The recombinant virus expressing the HA gene of H9N2 AIV, was generated using the strategy shown in Figure 1A, and designated as FAdV4-HA(H9). After blind passage, the RFP plaques indicating the recombinant virus FAdV4-HA(H9), and the EGFP indicating the parental virus FA4-EGFP, could be observed in the same visual field under the fluorescence microscopy after inoculated into the LMH cells, while no fluorescence was observed in the mock infected LMH cells (Figure 2A). The recombinant virus FAdV4-HA(H9) was then purified through limiting dilution and virus plaque assay. After several rounds of purification, the recombinant virus containing HA gene and the RFP expression cassette was successfully purified. To identify the insertion of HA in the recombinant virus FAdV4-HA (H9), the genome was extracted and further identified by sequencing and PCR. As shown in Figure 2B, two bands indicating the recombinant virus FAdV4-HA (H9) and parental virus FA4-EGFP, respectively, were detected before purification (lane 1). In contrast, the specific band indicating the recombinant virus was observed in the purified virus (lane 2), and the parental virus FA4-EGFP (lane 3) and wild-type FAdV-4 (lane 4) was set as control. The data demonstrate that the recombinant virus is successfully generated and purified.

### Identification of the expression of HA protein of H9N2 AIV in FAdV4-HA (H9)

**FIGURE 3:**
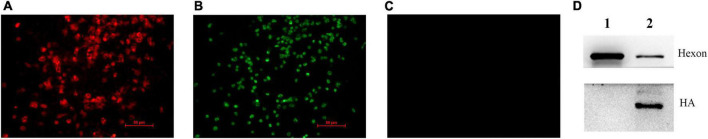
Identification of the expression of HA by the recombinant virus FAdV4-HA(H9). The expression of the HA protein in LMH cells infected with FAdV4-HA(H9) were detected by IFA **(A–C)** and western blot **(D)** using mAb 2G10 against HA of H9N2 and chicken serum against FAdV-4. **(A)** mAb 2G10 against HA of H9N2 was used. **(B)** Chicken serum against FAdV-4 was used. **(C)** LMH cells without infection. In the western blot, lane 1 indicated the cultured supernatant of the infected LMH cells; lane 2 indicated the cell lysate of the infected LMH cells. These experiments were performed twice with comparable results.

After purification, the RFP expression cassette was then removed by the Cre recombinase through the transfection of pcDNA3.1-Cre in LMH cells. After the RFP expression cassette was removed, the expression of HA of H9N2 AIV in the recombinant virus was further identified by IFA and western blot analysis. As shown in Figures 3A, B, the specific red fluorescence indicating the HA protein, and the specific green fluorescence indicating the FAdV-4 was detected under the same visual field in the LMH cells infected with FAdV4-HA(H9) in the IFA using the mAb 2G10 and the chicken serum against FAdV-4. Figure 3C was used as the negative control. In the western blot assay, as described in Figure 3D, the specific band of HA protein could be efficiently detected in the cell lysate of LMH cells infected with FAdV4-HA(H9), but not in the supernatant of the LMH cells infected with FAdV4-HA(H9). All these demonstrate the HA gene is successfully inserted into the genome of FAdV-4 as designed, and the HA gene is efficiently expressed in the cells infected with FAdV4-HA(H9).

### FAdV4-HA(H9) efficiently replicated and showed high stability in LMH cells

**FIGURE 4:**
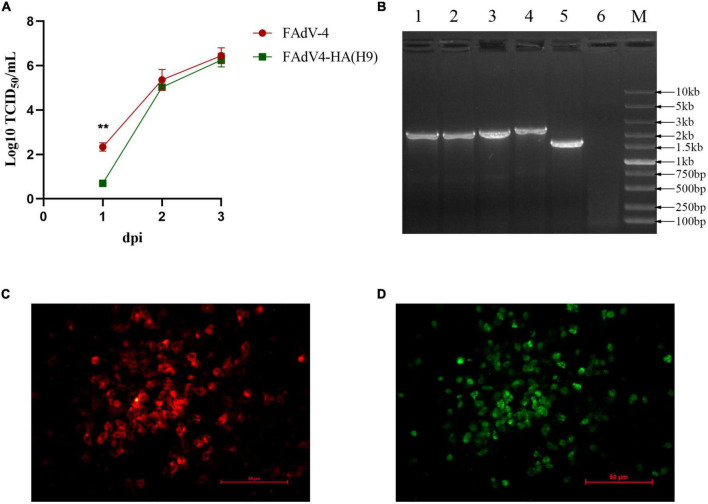
Growth curve and stability of the recombinant virus FAdV4-HA(H9). **(A)** LMH cells were infected with FAdV4-HA(H9) and FAdV-4 at the same dose, respectively, the viral supernatant collected from the infected LMH cells at the indicated time points were then titrated by TCID_50_. This experiment was done in triplicate and repeated twice. Statistical analysis in this experiment was performed with a Student’s *t*-test using GraphPad Prism 8 software. **(B)** PCR analysis of the recombinant virus FAdV4-HA(H9) at every 5 passage. lane 1-6 represent passage 5, passage 10, passage 15, parental virus FA4-EGFP, wild-type FAdV-4 and negative control, respectively. IFA analysis for the expression of HA in FAdV4-HA(H9) at passage 15 using mAb 2G10 against HA of H9N2 **(C)** and chicken serum against FAdV-4 **(D)**. These experiments were performed twice with comparable results.

To determine the growth curve of the recombinant virus FAdV4-HA(H9), the recombinant virus FAdV4-HA(H9) and wild-type FAdV-4 were respectively inoculated into LMH cells at the same dose, the supernatants from the infected LMH cells at different time points were collected and titrated. As shown in Figure 4A, the recombinant virus FAdV4-HA(H9) replicated at the same level with the wild-type FAdV-4, the peak titer of the recombinant virus could reach 10^6^ TCID_50_/ml at 3 dpi. To evaluate the stability of the HA gene in the recombinant virus FAdV4-HA(H9), the FAdV4-HA(H9) was continuously passaged for 15 passages in the LMH cells, and examined by PCR and IFA. As shown in Figure 4B, the band indicating the HA gene was detected in the recombinant virus, and no wild-type FAdV-4 were detected. We also performed sequence assay for the amplified HA gene from different passages, and did not find any additional mutation in HA gene in comparison with the HA gene of the passage 1 of FAdV4-HA(H9). For the IFA, the specific red fluorescence indicating the HA protein (Figure 4C), and the specific green fluorescence indicating the FAdV-4 (Figure 4D) were observed under the same visual field in the cells infected with the recombinant virus at passage 15. The IFA and PCR results suggest that HA gene is stably integrated and expressed in the recombinant virus FAdV4-HA(H9).

### FAdV4-HA(H9) was attenuated and could provide protection against H9N2

**FIGURE 5:**
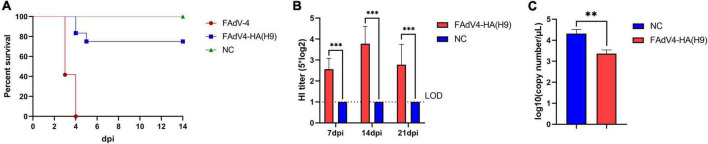
Chicken infection study with the recombinant virus FAdV4-HA(H9). Two-week-old SPF chickens were inoculated intramuscularly with the recombinant virus FAdV4-HA(H9) and wild-type FAdV-4, and monitored for 14 days. At 21 dpi, all the chickens survived were challenged with XZ491 and the oropharynx were collected at 5 dpc for viral loads. **(A)** Survival rates of SPF chickens after viral inoculation. **(B)** The HI titers against H9N2 AIV induced by the recombinant virus FAdV4-HA(H9) at the indicated time points. **(C)** Viral loads in the oropharynx from the chickens of group I [FAdV4-HA(H9)] and group III (NC) after challenge. Statistical analysis in this experiment was performed with a Student’s *t*-test using GraphPad Prism 8 software. ***p < 0.001.

To investigate the pathogenicity of the recombinant virus FAdV4-HA(H9), 2-week-old SPF chickens were inoculated with the same high dosage of FAdV4-HA(H9) and wild-type FAdV-4, and monitored for 21 days. As shown in Figure 5A, the chickens infected with wild-type FAdV-4 were all died within 4 dpi, while that infected with the recombinant virus FAdV4-HA(H9) only cause 25% mortality, and all the chickens in negative group were survived throughout the experiment. The HI titers of the serum were then evaluated, as shown in Figure 5B, the average HI titers against XZ491 was 31.1, 80, and 41.1 at 7, 14, and 21 dpi, respectively, and the highest HI titer could reach 160. However, the HI titers of the serum collected from the negative group were all less than 10. The survival chickens were then challenged with XZ491 at 21 dpi, the viral titers in the oropharynx at 5 dpc were determined by qRT-PCR. As shown in Figure 5C, the viral titers in the oropharynx of the chickens inoculated with the recombinant virus FAdV4-HA(H9) were significantly lower than those in the control group. All these demonstrate that the recombinant virus FAdV4-HA(H9) is attenuated and could provide protection against H9N2.

## Discussion

Since the outbreak of HHS caused by FAdV-4 was reported in Pakistan in 1987 (Cheema et al., 1989), the HHS gradually spread to the surrounding countries and even other continents (Toro et al., 1999; Roy et al., 2004; Gomis et al., 2006; Choi et al., 2012; Maartens et al., 2014; Li et al., 2016; Liu et al., 2016; Ye et al., 2016), resulting in great economic losses to the poultry globally. Currently, FAdV-4 has become prevalent worldwide, and there is an urgent need for the development of novel vaccines against FAdV-4, as well as the investigation of its pathogenesis. The construction of infectious clone through the reverse genetics, served as a traditional tool, has dramatically facilitated the study of fowl adenovirus. Using the infectious clones, the virulent factors and the vital sites responsible for the increased virulence of the novel FAdV-4 were identified (Zhang et al., 2018, 2021b; Liu R. et al., 2020), and the modification of Fiber enabled FAdV-4 with wider infection spectrum (Zhang et al., 2021a). On the other hand, various vaccines against the novel FAdV-4, as well as multivalent vaccines based on FAdV-4 were developed using reverse genetic technique (Pan et al., 2021; Zhang et al., 2021c,^2022^; Wang et al., 2022). However, all these methods need to clone the whole genome into a selective vector to construct the infectious clones, which were generally inefficient for generating recombinant FAdV-4.

In this study, a rapid and efficient method for the generation of recombinant FAdV-4 using the CRISPR/Cas9 and Cre-Loxp technique was first described. There are several approaches for targeted knock-in through CRISPR/Cas9 system, including homologous recombination (HR) (Bi et al., 2014; Zhang et al., 2017), non-homologous end joining (NHEJ) (Chang et al., 2017; Tang et al., 2018; Zai et al., 2022), microhomology-mediated end joining (MMEJ) (Nakade et al., 2014; Sakuma et al., 2016), and HMEJ (Yao et al., 2017). The HR requires a repair template with left and right homology arms (500–3,000 bp) that allowing precise insertion of exogenous genes. However, HR is generally considered inefficient since it only active during the late S/G2 phase (Saleh-Gohari and Helleday, 2004). Notably, NHEJ and MMEJ-based methods, with no homology arms or with microhomology arms (5–25 bp), enable the integration of exogenous genes into the genome at relatively high frequencies, however, NHEJ-based targeted integration introduced random directions and various indels at the junctions, and MMEJ-based integration exhibited low efficiency in cultured cells. Thus, the HMEJ-based method was used to improve integration efficiency. The length of homology arm is the most critical factor for targeted knock-in, as the efficiency increases as the homology arm length lengthened, and 600–1,000 bp is optimal length for high-level homology-directed recombination (Yao et al., 2017; Zhang et al., 2017). Therefore, the homologous arm at a length of 1000 bp was designed in our study. To further increase the knock-in efficiency, the donor template was then flanked with two sgRNAs recognition sequence, as previous studies demonstrated, the donor template released by the sgRNA could lead to a 2–5 folds increase in HR efficiency compared to the circular donor plasmid (Zhang et al., 2017). We also tried HR method without the RFP expression cassette and NHEJ method for the generation of recombinant FAdV-4. However, both methods showed low efficiency when generating the recombinant virus expressing exogenous genes, which clearly demonstrated the high efficiency of the HMEJ method in our study.

It should also be mentioned that the procedure for screen and purification of the recombinant FAdV-4 was very simple and highly efficient in the CRISPR/Cas9 and Cre-Loxp system used here. In this approach, the recombinant virus FA4-EGFP with the expression of EGFP-Fiber-2 fusion protein was used as a template virus to replace the wild type FAdV-4, and the plasmid carrying with the homologous arms, Fiber-2 tail of FAdV-4, exogenous gene, RFP expression cassette flanked with Loxp, and sgRNAs recognition sequence as a donor plasmid. Therefore, the plaque of the recombinant FAdV-4 with foreign gene rescued in LMH cells would be easily found through the observation of the RFP, which was different from the template virus with the EGFP. Through the pickup of the plaque with RFP, the recombinant FAdV-4 with foreign gene could be efficiently purified by limit dilution, and the purity of the purified recombinant FAdV-4 could also be easily identified under fluorescence microscopy. The RFP in the purified recombinant FAdV-4 with foreign gene could be further efficiently deleted through the Cre recombinase. Through this novel approach, we successfully generated a recombinant virus FAdV4-HA(H9) with the expression of HA of H9N2 AIV. As shown in Figure 3D, the expression of HA protein of H9N2 AIV mainly was located in the lysate of LMH cells infected with FAdV4-HA(H9), but hardly detected in the supernatant in the infected LMH cells, suggesting that the HA protein expressed by FAdV4-HA(H9) might not be inserted into the viral particle. Notably, the infection of FAdV4-HA(H9) with high dose of 10^5^ TCID_50_ only caused 25% mortality in 2-week-old chicken, whereas the wild type FAdV-4 with the same dose resulted in 100% mortality within 4 dpi. Since *fiber-2* gene plays viral roles in the pathogenesis of FAdV-4, the lack of *fiber-2* gene in FAdV4-HA(H9) may contribute to the attenuated phenotype of FAdV4-HA(H9) in comparison with the wild type FAdV-4. The preliminary challenge study in these survival chickens infected with FAdV4-HA(H9) demonstrates the efficacy of the inoculation of FAdV4-HA(H9) against the challenge of H9N2 strain XZ491 (Figure 5C). The systematic immune-protective study with different vaccination doses of FAdV4-HA(H9) will be evaluated in future.

In conclusion, it is the first demonstration of a rapid and efficient approach for the generation of *fiber-2*-edited attenuated recombinant FAdV-4 through the CRISPR-Cas9 and Cre-Loxp system. The novel recombinant virus FAdV4-HA(H9) generated in this study can serve as a potential vaccine candidate for the control and prevention of both FAdV-4 and H9N2 AIV. Currently, we have used this strategy to efficiently generate several other recombinant FAdV-4 viruses, which expressing the HN of Newcastle disease virus, the Fiber-2 of duck adenovirus 3, the VP1 of chicken infectious anemia virus, and the VP2 of infectious bursal disease virus, respectively (Data not shown). All these constructed recombinant FAdV-4 viruses further highlight the feasibility of the strategy developed here for generation of *fiber-2*-edited attenuated recombinant FAdV-4 vector carrier. Moreover, the technique for constructing recombinant FAdV-4 developed in this study is also an promising tool for the manipulation of genomes, as well as studies on viral gene function and virus-host interactions.

## Data availability statement

The original contributions presented in this study are included in the article/supplementary material, further inquiries can be directed to the corresponding authors.

## Ethics statement

The animal experiments were performed in accordance with the “Guidelines for Experimental Animals,” which was approved by the Animal Care and Use Committee of Yangzhou University (Yangzhou, China).

## Author contributions

YG, QX, and JY designed the study, analyzed the data, and wrote the manuscript. YG, YC, XC, HJ, HL, TL, ZW, QX, and ZX performed the experiments. JY, HS, and AQ analyzed the data. JY and QX supervised the experiments and acquired the research funds. All authors participated in the development of this manuscript, contributed to the article, and approved the submitted version.

## Footnotes

1
http://crispor.tefor.net/
